# Covid-19-associated pulmonary aspergillosis in mechanically ventilated patients

**DOI:** 10.1186/s13613-024-01268-4

**Published:** 2024-03-01

**Authors:** Guangting Zeng, Linlin Wang

**Affiliations:** 1 Department of Pharmacy, The First People’s Hospital of Chenzhou, Xiangnan University, Chenzhou, China; 2 Department of Pharmacy, Cancer Center(Xiamen), Fudan University, Xiamen, China

Dear editor,

We read with great interest the multicenter study conducted by Desmedt et al. that detailed the incidence and outcomes of COVID-19-associated pulmonary aspergillosis (CAPA) in mechanically ventilated patients [1]. Their research revealed probable CAPA is a rare but serious complication of severe COVID-19 requiring mechanical ventilation.Therefore, neither routine screening for aspergillus disease nor preventive treatment is supported. Here, we would like to highlight some points that deserve discussion and serious attention.

Invasive aspergillosis has become an important superimposed infection among COVID-19 patients and is associated with poor outcomes. The prognosis of severe CAPA is poor, with a fatality rate of 22.2-71.4% [2]. However, due to factors such as population heterogeneity, screening strategies, and definitions used, the incidence of CAPA varied widely, ranging from 2.4–34.3% [3]. This has also led to controversy over whether routine screening and preventive treatment for aspergillus should be carried out in severely ill patients.

In Desmedt et al. ‘s study, only 18 patients (2.5%) met the criteria for probable CAPA.The low incidence may be related to population characteristics and screening strategies. First, the study’s population included COVID-19 patients who required mechanical ventilation, but the proportion of immunocompromised people was low (16%) and only 8.2% of patients with active blood or solid malignancies. A recent systematic review and meta-analysis by Francesca Gioia and colleagues confirmed that hematological malignancies are associated with CAPA and are a high risk factor [4]. Two meta-analyses showed that high doses of corticosteroids and treatment of COVID-19 with interleukin-6 inhibitors such as tocilizumab were also associated with a significantly increased incidence of CAPA [4, 5]. Approximately two-thirds of the patients in this study received corticosteroid therapy, the dosage of which was not described, while only four (0.57%) patients were given an IL-6 antagonist. Second, the study was conducted in 15 intensive care units, and only two centers actually performed routine screening for aspergillus, and these two centers screened half of the probable CAPA (9/18).

Given the extremely poor prognosis for patients with CAPA, there remains a need to identify risk factors and routinely screen high-risk populations and use targeted prevention or early empiric treatment to prevent CAPA and reduce mortality.

## Data Availability

Our manuscript has no associated data.

## Declarations

### Completing interests

On behalf of all authors, there is no completing interest.
